# Note Onset Deviations as Musical Piece Signatures

**DOI:** 10.1371/journal.pone.0069268

**Published:** 2013-07-31

**Authors:** Joan Serrà, Tan Hakan Özaslan, Josep Lluis Arcos

**Affiliations:** IIIA-CSIC, Artificial Intelligence Research Institute, Spanish National Research Council, Bellaterra, Barcelona, Spain; University of Adelaide, Australia

## Abstract

A competent interpretation of a musical composition presents several non-explicit departures from the written score. Timing variations are perhaps the most important ones: they are fundamental for expressive performance and a key ingredient for conferring a human-like quality to machine-based music renditions. However, the nature of such variations is still an open research question, with diverse theories that indicate a multi-dimensional phenomenon. In the present study, we consider event-shift timing variations and show that sequences of note onset deviations are robust and reliable predictors of the musical piece being played, irrespective of the performer. In fact, our results suggest that only a few consecutive onset deviations are already enough to identify a musical composition with statistically significant accuracy. We consider a mid-size collection of commercial recordings of classical guitar pieces and follow a quantitative approach based on the combination of standard statistical tools and machine learning techniques with the semi-automatic estimation of onset deviations. Besides the reported results, we believe that the considered materials and the methodology followed widen the testing ground for studying musical timing and could open new perspectives in related research fields.

## Introduction

Music is an outstanding means of mankind's emotional expression [1]. In western art music, it is known that such emotional expression owes to a great deal to the way performers interpret a given piece or composition, beyond what is written in the printed score [2], [3]. Indeed, systematic and significant deviations from the strict rendition of the piece are an essential aspect of music performance [4], [5]. Moreover, apart from emotional expression, such deviations are believed to strongly contribute to the comprehension of the musical message [3], [6]. They are so common that the mere presentation of music as notated in the score sounds too mechanical and highly unmusical to us (cf. [7]–[10]). As Cook puts it [8], “the way performers shape notes brings music to life”.

Expressive deviations rely on the manipulation of sound properties such as pitch, timbre, dynamics, and timing [4], [5]. Timing is often considered to be the most important expressive resource, and is perhaps the only variable over which any performer has practically complete control, regardless of the instrument used [11]. Timing generally refers to variations in the temporal organization of musical events introduced by a performer as compared to the strict adherence to tempo and notated score values. Such variations can relate to different temporal aspects such as note duration (interval timing; see, e.g., [12]) or note onset delay/anticipation (event-shift timing; see, e.g., [13]), and can be represented in different ways [14]. Research on timing deviations has a long history, dating back to the beginnings of the twentieth century (for pointers to such early works we refer to [4]). Overall, the wealth of existing literature confirms that performers make “systematic and significant deviations from strict metricality” but, at the same time, indicates that “it is hard to make generalizations about the nature of [such] deviations” [11].

In the literature we find different views on the origin of timing deviations. There is evidence that timing deviations help the listener to clarify phrasing [15]–[17], metrical accents [18], musical form [19], and harmonic structure [20], [21]. Complementarily, different note patterns or groups exhibit some common timing “tendencies” [11], possibly affected by tempo transformations [22]. All these works point towards musical structure as a source for timing deviations, constituting the basis of the so-called generative approach [6]. However, to the best of our knowledge, as yet there is no systematic, compelling, and large-scale study in this direction (i.e., involving multiple pieces, performers, instruments, styles, and epochs). Moreover, timing deviations might not arise solely from music structure. It has been also shown that they can be idiosyncratic of a performer's style [19], [20], [23], to the point that machines can identify such performers using automatically-extracted timing information [24], [25]. Emotional expression is also assumed to play an important role [1], [2]. Besides, we also find the so-called perceptual hypothesis [26], in which some observed variations would be due to functional constraints of the auditory system. This way, some time intervals would be heard shorter and thus played longer as a phenomenon of perceptual compensation [26]. Additionally, some timing deviations may be shaped in accordance to patterns of biological motion [27] or instrument-related motion [4], [5]. Notice that all the previous references assume that timing deviations are, to a large extent, under the control of the performer and, thus, introduced voluntarily. Nonetheless, one could always attribute timing deviations, to some extent, to random temporal variability exogenous to the interpretation of the piece [27], originating from human internal clock and motor noise [28].

In this article, we explore a complementary view on the structural origin of timing deviations. In particular, we cast timing deviations as being characteristic of a given composition, up to the point of allowing the automatic identification of the musical piece the recording belongs to. To validate this hypothesis we consider onset deviation sequences or n-grams, i.e., the succession of temporal anticipations or delays for each note attack. The choice of this event-shift timing representation is motivated by the highly percussive nature of the instrument being considered: classical guitar. Classical guitar recordings represent an interesting test corpus, as almost no studies on timing deviations consider this instrument. One exception that does deal with general expressiveness in guitar recordings is [29] (this work also states a lack of research with expressiveness and emotional performance with this instrument and refers to some of the early works on such a general topic). Noticeably, guitar recordings facilitate note onset detection, as prominent attack times are present for almost all notes. To obtain accurate attack times we rely on a score-synchronized, semi-automatic approach to onset detection (see Materials & Methods, MM). This allows us to go from the analysis of single, experiment-specific performances to medium-scale real-world music collections. We consider 100 professional/commercial performances of 10 well-known classical guitar pieces of different styles, spanning different epochs, and with some performers interpreting different pieces.

By formulating our hypothesis as a classification problem and, thus, within a strong statistical framework [30]–[32], we gain objective and quantitative evidence for the structural, piece-dependent nature of onset deviations. To show that the predictive power of onset deviation sequences is generic and not biased towards a specific classification scheme, we consider 7 basic algorithms exploiting five different machine learning principles [30]–[32]: decision tree learning, instance-based learning, linear regression, Bayesian learning, and support vector machines. Specifically, we use nearest neighbor algorithms with Euclidean and dynamic time warping distances (NN-E and NN-D, respectively), classification and regression trees (Tree), a naive Bayes Gaussian classifier (NB), a logistic regression model (LR), and support vector machines with linear and Gaussian kernels (SVM-L and SVM-R, respectively). We additionally consider a random classifier as a baseline. To evaluate identification performance we employ standard out-of-sample cross-validation accuracies [30]–[32], and to evaluate statistical significance we depend on the power of the Wilcoxon signed-rank test [33] with Holm-Bonferroni adjustment [34] (see MM).

## Results

**Figure 1:**
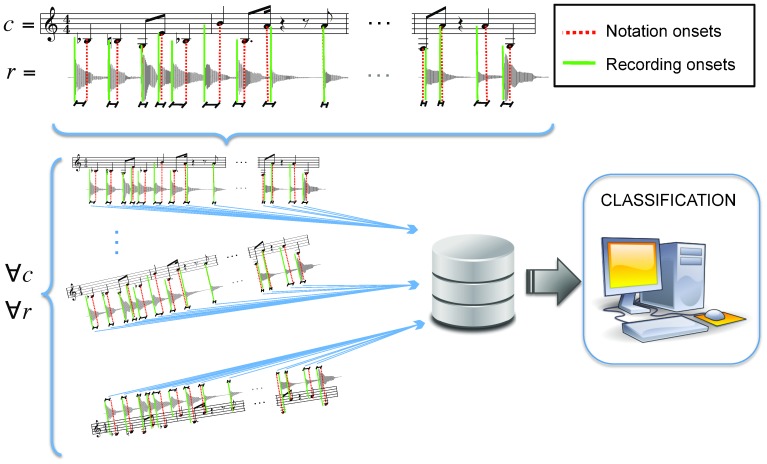
Methodology overview. The difference 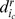 between notation and recording onsets is computed, and sequences of such differences 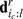 are used to train a classifier and to perform an out-of-sample cross-validation. Note indices 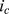 are randomly selected for each composition, 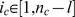, keeping the same 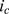 for all recordings of the same composition (see MM). The length of the sequences 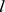 is kept as a parameter in our evaluation.

To compute onset deviations from the score we follow a semi-automatic approach that yields a plausible placement of note attacks [35]. In particular, we combine standard onset detection algorithms for music signal processing [36]–[38] with a manual synchronization of the score measures. With the latter, we can correct potential errors in the automatic onset detection stage and, furthermore, determine a ‘theoretical’ temporal onset location corresponding to a straight, mechanical rendition of the piece. The actual onset locations are then subtracted from the corresponding notated locations, as if we were measuring temporal differences from the score (Fig. 1). More specifically, the onset deviation for the 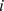-th note of the 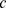-th composition in the 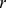-th recording is obtained by.
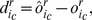
(1)where 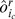 is the temporal location of the 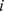-th note onset according a straight and manually synchronized rendition of composition 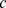 (the one to which 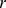 belongs) and 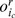 is the actual temporal location of such onset in the 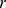-th recording. Here, we express all temporal variables in seconds. However, for comparison purposes, the full sequence corresponding to a composition with 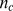 notes, 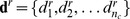, is normalized so that it has zero mean and unit variance, thus leading to dimensionless units. After normalization, subsequences of 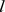 consecutive onset deviations, 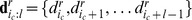, are used as n-gram features for classification, taking 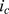 by uniform sampling from all possible notes of the piece (thus we guarantee the same 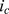 for all renditions of the same composition 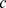). This sampling is performed 100 times, yielding a sufficiently representative case base from all possible combinations of sequences coming from different pieces. Further details of the followed methodology can be found in MM.

In pre-analysis, we checked whether the magnitude of the obtained onset deviations could be inferred from basic score notation. The results suggested that the considered onset deviations are quite independent of their associated relative note duration, expressed with relation to the beat (e.g., 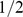 beat, 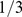 beat, 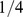 beat) or their associated pitch interval size, expressed in semitones (e.g., 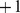 semitone, 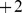 semitones, 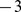 semitones). Specifically, non-significant correlations were found (Figs. S1 and S2). Overall, we found no compelling evidence of the relation between onset deviations and the most fundamental short-time score elements, i.e., the single notes. Apart from that, and as a side note, we also observed that the distributions of onset deviations generally do not conform to a standard Gaussian distribution (MM and Fig. S3A). This aspect, if further confirmed, would differentiate (largely voluntary) onset deviations from involuntary beat fluctuations in human rhythm tapping [9], which were well approximated by standard Gaussians. As a further side note, we found some qualitative indication of long-range correlations in 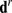 (MM and Fig. S3B). In the case that long-range temporal correlations existed, akin to the ones already observed in rhythm notation [39] and in involuntary rhythm tapping fluctuations [9], onset deviations performed by experienced musicians could be cast as memory processes [40], and thus may have the potential to contain non-trivial information of their context.

**Figure 2:**
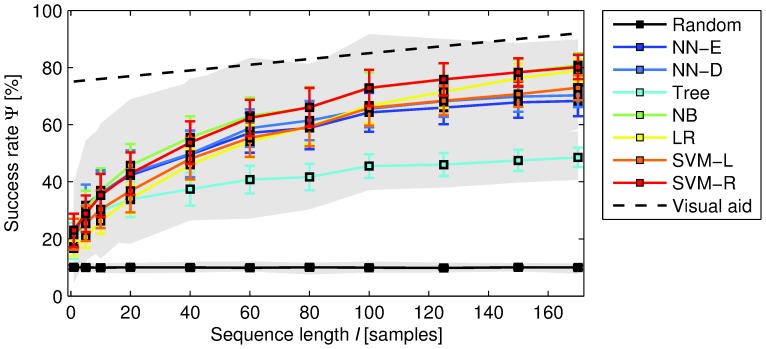
Classification accuracy as a function of the length of the onset deviation sequence. The error bars correspond to the standard deviation and the shaded area denotes the range of all possible values (including minimum and maximum). The visual aid corresponds to a straight line of the form 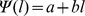, where 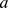 is the intercept, 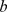 is the slope of the straight, and 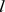 is the sequence length. In the plot 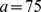 and 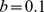.

**Figure 3:**
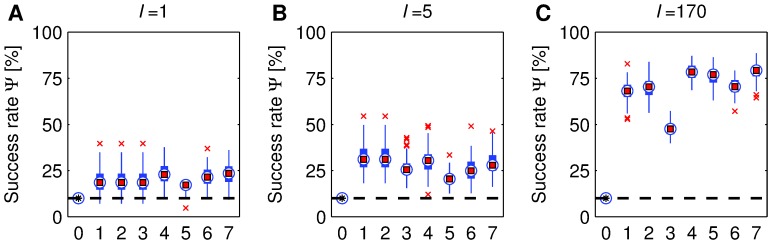
Box plot of classification accuracies using different sequence lengths. These are 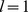 (A), 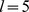 (B), and 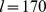 (C). The labels in the horizontal axes correspond to classification algorithms: Random (0), NN-E (1), NN-D (2), Tree (3), NB (4), LR (5), SVM-L (6), and SVM-R (7). In all plots, all accuracies are statistically significantly higher than the random baseline (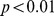, see MM).

We now leave the preliminary analysis and its related conjectures and concentrate on our main contribution, i.e., assessing whether onset deviations have some predictive power of the composition being interpreted. If we plot the classification accuracies 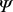 as a function of 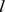 we see that all classifiers perform on a similar range, with NB and SVM-R generally achieving the best accuracies (Fig. 2). As expected, NN-E and NN-D perform relatively similarly, thus indicating that no strong sequence misalignments are present, thanks to the aforementioned semi-automatic measure-based synchronization between score and recordings (see also MM). Trees achieve the lowest accuracies and seem to have some difficulties in learning from the considered sequential information. Nevertheless, for 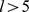, all obtained accuracies lie far beyond the random baseline, always increasing with 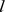 (Fig. 2). Importantly, we see that statistically significant accuracies can be reached with very short sequences 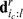 (Fig. 3). Specifically, it turns out that a single sample 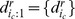 is sufficient to characterize a piece statistically significantly beyond the random baseline, but with a low accuracy (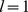, Fig. 3A). This difference increases with 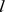, until no single accuracy across 100 trials goes below the ones achieved by the baseline (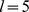, Fig. 3B). Obviously, the longer the deviation sequence, the more powerful the discrimination between compositions (e.g., 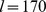, Fig. 3C). Shuffling 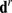 results in classification accuracies that are almost constant with 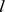 (Fig. S4) and, within the range of those achieved for 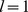 above. This indicates a temporal dependency between onset deviations, and that this dependency is crucial for the task at hand.

**Figure 4:**
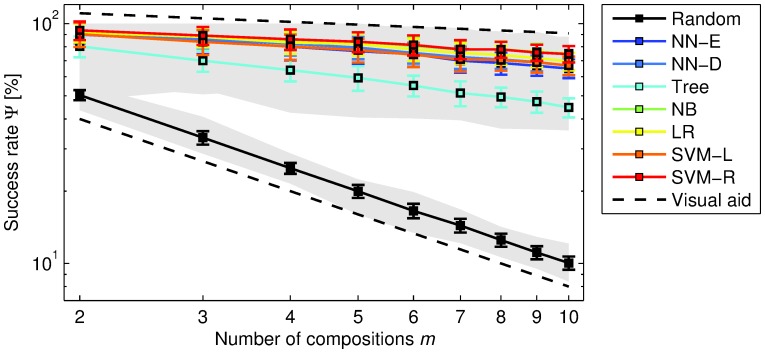
Average classification accuracy as a function of the number of compositions. Results obtained using a sequence length 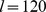 (for 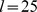 see Fig. S5). The error bars correspond to the standard deviation and the shaded area denotes the range of all possible values (including minimum and maximum). The visual aids correspond to a power law of the form 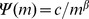, where 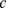 is a constant, 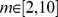 is the number of compositions, and 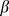 is the power law exponent. The upper one is plotted with 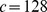 and 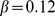, and is associated with classification accuracies. The lower one is plotted with 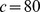 and 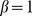, and corresponds to the random baseline. Notice that the exponent associated with classification accuracies is much smaller than the one for the random baseline, which suggests that the absolute difference between the two increases with the number of considered compositions and, therefore, with the size of the data set.

**Figure 5:**
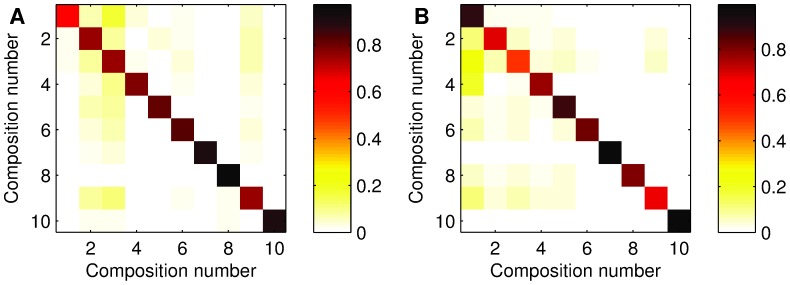
Confusion matrices for two different classifiers. These are NB (A) and SVM-R (B). The color code indicates average accuracy per composition (the higher, the darker). Compositions 7, 8, and 10 seem to be generally well-classified. For NB, compositions 2 and 3 attract many of the confusions while, for SVM-R, composition 1 takes that role.

To check whether the predictive power of onset deviation sequences is robust with respect to the size of the music collection, we can plot the accuracies 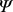 as a function of the number of compositions 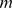 (Fig. 4). With this we observe that the obtained accuracies decrease at a much lower rate than the ones provided by our random baseline, independently of 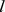 (see also Fig. S5). This shows that onset deviations can be a reliable predictor of a musical piece. Additionally, we confirm that accuracies are balanced across compositions, with no exceptional confusion between pairs of them (Fig. 5). In fact, we see that such confusions depend on the classifier. This suggests that a specific confusion may not be due mostly to the onset deviations themselves and, furthermore, that a strategy based on an ensemble or a combination of classifiers [31], [32] could potentially increase the overall accuracy. As our objective here is more focused on showing the predictive power of onset deviations rather than achieving very high accuracies on a music classification task, we leave the above strategy for future work.

Finally, it is worth mentioning that we also studied the classification accuracies using sequences of note durations relative to the beat, instead of note onset deviations. We followed exactly the same procedure as for the onset deviations but substituting this information by the relative note durations as written in the score (we made 10 exact replicas from such information in order to emulate having 10 recordings of each composition, see MM). The results showed that the onset deviation accuracies are similar, or in some cases even higher, than the note duration ones (Fig. S6). Interestingly, the best performing classifiers, NB and SVM-R, were also the ones where such a difference was more clearly observable. Notice that, as mentioned, relative note durations were found to be uncorrelated to onset deviations (Figs. S1 and S2).

### Limitations of the Study, Open Questions, and Future Work

The reported results can be argued to provide quantitative support for a generative origin of timing deviations, i.e., that these respond to the structure of the musical piece and its psycho-perceptual consequences for interpretation. As mentioned in the Introduction, there is evidence that musical aspects such as tempo, phrasing, metrical accents, musical form, and harmonic structure can determine timing deviations. In our experiments, mixing different compositions and their interpretations, we found scarce evidence for the dependence of onset deviations on individual score elements (specifically, of note intervals and relative durations). However, this does not preclude other score information like the aforementioned musical aspects having a direct influence on onset deviations. The fact that onset deviations perform similarly or slightly better than relative note durations, combined with the fact that the former were independent and uncorrelated to the latter, also suggests that onset deviations encapsulate information that goes beyond duration/temporal aspects of the score. Additional medium- or large-scale quantitative studies with real-world commercial recordings of classical guitar could provide more insight into this question. An alternative plausible hypothesis for the obtained results would be that onset deviations were so specific to the musical composition, that deterministic rules inferred from a pool of compositions could not be generalized to cover all the variability in the pool. As existing research suggests, this hypothesis cannot be completely ruled out (cf. [11]). Thus, one should also be open to the possibility that timing deviations encapsulated some contextual aspects specific to the composition but not related to the score (e.g., composer-specific performance rules, historical performance considerations, etc.). Regarding this latter hypothesis, it could perhaps be interesting to replicate the experiments carried out here considering cover songs or jazz versions, as these retain the essence of the original composition while usually introducing important changes in timbre, harmony, tempo, or rhythm [41] (although semi-automatic onset extraction could be more involved).

As our study was not designed to do so, the reported results only provide weak evidence regarding the additional hypotheses on the origins of timing deviations mentioned in the Introduction. It is true that the considered music collection contains a number of recordings of different pieces by the same performer. Hence, if performer-specific deviations dominated the raw onset sequences, one would expect much worse piece identification accuracies, as recordings would tend to cluster around performers and not around pieces. However, the lack of a sufficient number of performers having more than one recording in the considered collection seriously challenges clustering across performers and does not allow any strong claim regarding this hypothesis to be made (it is worth mentioning nonetheless that, as some works indicate [19], [24], [25], performer-specific aspects may be better studied after subtracting a global, average performance template like the one we consider here). A similar argument holds for emotion-based hypotheses. Assessing the biological or instrument-related motion hypothesis would require a different collection containing recordings of the same piece played with different instruments (perhaps the cover songs or jazz versions mentioned above). In the present study, we wanted to focus on the classical guitar, as this is an almost unexplored area. Finally, our results suggest that randomness is a minor component of the considered onset deviations, reinforcing their largely voluntary nature. Indeed, if noise were very present in the considered onset deviations, we would not be able to achieve the reported classification accuracies. A precise quantification or estimation of the amount of noise in timing deviations is, nonetheless, out of the scope of the present study (it may moreover have a lot of dependencies: instrument, genre, performance difficulty, etc.).

Regarding the reported preliminary assessments, we are aware that many further improvements can be done, specially for the case of long-range correlations. A complete characterization of note onset deviations, their relation to all possible score elements, their distribution, and their long-range correlations is beyond the scope of the present study. Nonetheless, we believe our assessments are some of the necessary first steps towards these goals and could motivate future research and discussion (for instance, the fact that onset deviations do not conform to a standard Gaussian distribution could lead researchers in machine-based music rendering to explore other distributions that could result in a more plausible listening experience). Hence, we opt to include, link to some literature and briefly explain such assessments.

### Conclusions

In summary, the obtained results show (a) that onset deviation sequences are a powerful predictor of the musical piece being played, (b) that they are at least as powerful as direct music score information corresponding to relative note durations, if not better, (c) that such predictive power is robust to classification scheme choices, to the size of the considered data set, and to the length of the considered sequences, (d) that even very short sequences provide statistically significant accuracies, and (e) that temporal dependencies between onset deviations are key to obtaining such accuracies. Moreover, we quantify how the length of onset deviation sequences and, to a lesser extent, the size of the data set, impact classification accuracy. Some additional preliminary experiments are reported. In particular, our results show non-significant correlations between onset deviations and relative note durations or pitch intervals, and indicate that onset deviations do not obey a Gaussian distribution. Finally, we discuss existing open issues and some of the limitations of our study, while linking our findings to the existing literature.

As a main objective, this article wants to provide a new and fresh view on the topic of music timing variations. We believe that by taking quantitative medium-scale approaches, considering real-world commercial recordings, and different instruments apart from piano is a necessary step towards a better understanding of it. Here, the focus is on the utility of onset deviation sequences as musical piece signatures, and on the predictive power of those sequences. Hence, our main contribution relies on showing this predictive power and studying it under different temporal windows, even very short ones. With this in mind, the results found are encouraging and open new research perspectives. We hope future work will bring more evidence on the connection between musical pieces and the onset deviations extracted from their performances.

## Materials and Methods

### Music Collection

In our music collection we have 10 different compositions, and each composition is performed by 10 different guitarists, thus yielding a total of 100 recordings. However, some performances of different compositions have been interpreted by the same musician. In total, we have 82 different guitarists, with some of them playing between 2 and 5 pieces. The collection includes well-known guitarists such as Andrés Segovia, John Williams, Manuel Barrueco, Rey de la Torre, Robert Westaway, and Stanley Myers. In order to encompass different epochs, we chose compositions spanning four different periods: baroque, classical, romantic, and modern (Table S1). Recording years go from 1948 to 2011, and the number of onsets per score measure varies between 1 and 16. A table relating compositions, recordings, and performers is provided (Table S2).

### Semi-automatic Onset Detection

In music signal processing, different techniques of varying complexity for automatic onset detection exist [36], [37]. These usually work on the time domain, the frequency domain, or both [37], [38]. Due to the difficulty of the task, it is not expected that a single method or parameter combination will work for all possible specific cases [37]. Therefore, we needed to choose the correct onset detection algorithm according to our needs, and tuned its parameters appropriately for the data at hand. In our case, we considered the 7 available algorithms in the Aubio library (http://aubio.org) [38]. To choose one of the algorithms and its best-fitting parameters we implemented particle swarm optimization [42] and ran it over an independent, out-of-sample set of 12 classical guitar audio files with manually annotated onsets [35]. The best performing combination was found to be the Kullback-Leibler algorithm [43] with a window length of 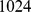 samples, a hop size of 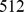 samples, a peak-picking energy threshold of 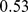, and a silence threshold of 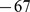 dB (the sample rate was 44.1 KHz; for further explanations we refer to the Aubio documentation (http://aubio.org/doc/onsetdetection_8h.html) and [38]).

After detecting the onsets in our collection using the algorithm and parameters above, we implement an additional onset validation step. For that, we first manually annotated the score measure positions of all recordings in our collection. This way, we could unambiguously synchronize each measure in the audio file with the corresponding measure positions in the written score. The reference onset positions 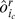 were then assigned by distributing the onsets between each measure according to strict score notation (Fig. S8). Additionally, we checked whether there were missing onsets. If a score onset 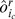 did not match an audio onset 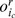, we imputed the temporal location corresponding to 7 milliseconds before the highest audio signal magnitude (absolute values) closest to 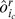 and within a short-time window (Fig. S8). We used a window centered at 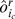 whose length corresponded to the 90-th percentile value of the composition's note durations (the 7-millisecond offset was manually determined by visual inspection of a small subset of the real data).

To check the accuracy of the obtained 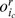, we manually validated 223 onsets, randomly sampled from the whole data set. Specifically, we annotated the temporal difference between what we considered to be the true onset location and the one determined by our approach (Fig. S7A). The vast majority of the inspected onsets were at their correct locations. Using a threshold evaluation strategy to determine the percentage of correct onset placements [38], we estimated that only a 6.7% of them were not placed on the exact location they should be. This number drops to 2% if we consider a threshold of 150 milliseconds (Fig. S7B).

After extracting onset positions 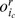, onset deviations are computed as in Eq. 1, obtaining a sequence 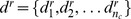 for a composition with 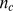 note onsets. Notice that, as mentioned in the Introduction, this is an event-shift representation of timing. Notice furthermore that, due to the manual synchronization of each score measure with the audio signal, the first onset of each measure would result in 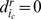, thus losing several meaningful onset deviations. To alleviate this problem we consider a 4-measure window synchronization, and average the onset deviations 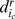 obtained when moving this window in steps of one measure (i.e., the final 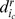 in the 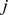-th measure is obtained by averaging the four 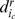 obtained by synchronizing the beginning of measures 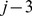 and 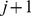, 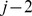 and 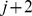, 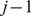 and 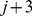, and 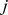 and 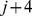). Thus, with the exception of the onsets at the beginning and end of the piece, 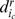 would be obtained as the average over four deviation values (we however made a further refinement and avoid the extremes, i.e., the maximum and minimum values, and compute the average between the two central ones). The raw onset deviations for the considered recordings can be found online (http://www.iiia.csic.es/~tan/downloads/2013_OnsetDeviations_Data.tar).

### Preliminary Side Checks

All compositions provided similar numbers for the statistics of raw onset deviation values 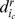 (Table S3). Additionally, we manually confirmed that maximal anticipations/delays generally corresponded to full cadences, usually ritardandos found in piece endings or strong structural locations (cf. [19]–[21], [25]). For instance, in the middle of the twenty-first measure of C02 (J.S. Bach, BWV 1007), for all performances, we observed a long pause between 0.5 and 1 seconds, which does not correspond to any existing annotation in the written score. Also, in C03 (A. Barrios, La Catedral–Prelude), the notes corresponding to the melody in the arpeggios are significantly delayed in most of the performances.

As a separate preliminary check, we ran some tests in order to assess the nature of the distribution of the samples in 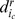. First, we checked whether such distribution could be assumed to be a standard Gaussian distribution
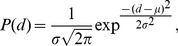
(2)where 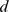 here stands for a single onset deviation value and 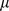 and 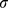 correspond to the mean and standard deviation of all 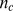 values in 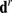, respectively. For each recording we ran an Anderson-Darling test [33] for the null hypothesis that 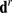 was drawn from a Gaussian distribution. The Anderson-Darling test is known to be one of the most powerful statistical tools for detecting most departures from normality [44]. Under such test, the null hypothesis was rejected for 93 of the 100 recordings at a significance level of 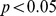 and for 88 of the 100 recordings at 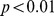. Visual inspection of the data also gives us a qualitative confirmation of this result (Fig. S3A).

Next, as a further separate preliminary check, we wanted to assess whether we could find some indication of long-range correlations in 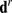. For that, inspired by [9], [39], we performed a small qualitative investigation of whether a power law could explain the power spectral densities 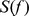 obtained from 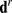. In particular, we considered whether
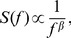
(3)where 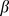 is the power law exponent. A visual inspection of individual linear fits to the power spectral densities 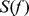 obtained from different recordings suggests the possibility of a power law (Fig. S3B). Noticeably, the fits yielded exponents 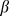 in the ranges provided by [9]. Since, as mentioned, we only wanted to have an impression of the behavior of 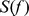, we did not pursue more robust power law fitting strategies such as the ones followed in our previous work [45], [46] or elsewhere [47], nor did we consider more advanced techniques for determining the existence of long-range correlations such as the ones employed, e.g., in [9], [39].

### Feature Extraction

The features we use as input for classification are normalized onset deviation subsequences or n-grams. First, the entire sequences 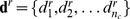 for each recording 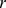 are normalized to have zero mean and unit variance, 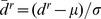, where again 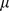 and 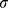 correspond to the mean and standard deviation of all 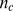 values in 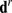. Next, for each composition 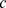, the one to which the 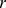-th recording belongs, an integer note index 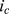 is uniformly chosen, 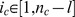. This, together with the predefined sequence length 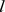, determines a subsequence 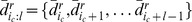. The final data 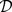 that serves as input for the classifier consists of the union of n-gram feature sequences plus the composition labels across all recordings. Formally,
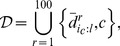
(4)where 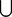 denotes the union operator and, as mentioned, 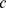 indicates the composition index of the 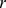-th recording. Notice that, due to the random choice of 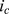 and the fact that 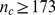 (Table S1), the 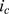 for each composition might be different. However, notice also that 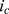 is the same for every recording of composition 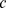. Hence, the same subsequence position is taken for all recordings of a composition.

Apart from onset deviations, some of the performed experiments consider other information from the score. This is the case for pitch intervals and relative note durations. Pitch intervals, which we denote by 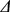, are expressed in semitone differences between consecutive notes (e.g., 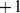 semitone, 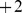 semitones, 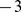 semitones). Relative note durations, which we denote by 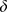, are taken as the written note duration with respect to the beat (e.g., 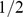 beat, 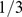 beat, 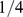 beat). In the latter case, for classification, we compute the 10 different sequences (one for each composition) and then produce 10 copies of each in order to emulate 100 performances. The rest of the process is the same as explained above except that, in the normalization step, we replace the mean 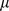 by the mode of the distribution. We believe this is a more sensible approach, as the distribution of relative note durations is discrete and often discontinuous (see Fig. S1). Notice that, in the case of relative note durations and in contrast to onset deviations, there will be no differences between performances. This makes relative note durations a very strong adversary against which the predictive power of onset deviations can be compared.

### Classification

We cast the problem of identifying the piece from its onset deviations as a 10-class classification problem [30]–[32]. To show that the predictive power of the considered feature sequences is generic and not biased towards a specific classification scheme, we employ basic algorithms exploiting five different machine learning principles [30]–[32]: decision tree learning, instance-based learning, logistic regression, probabilistic learning, and support vector machines. The algorithm implementations we use come from scikits-learn (http://scikit-learn.org) version 0.10 and, unless stated otherwise, their default parameters are taken. Since our focus is on assessing the predictive power of onset deviation sequences rather than obtaining the highest possible classification accuracies, we make no tuning of the classifiers' parameters. In total we use 7 algorithms [30]–[32] plus a random classifier:

- NN: 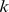-nearest neighbor classifier. We use the Euclidean distance (NN-E) and dynamic time warping dissimilarity (NN-D). For dynamic time warping we use a standard implementation with a global corridor constraint of 10% of the sequence length [48]. The number of neighbors is arbitrarily set to 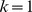.
- Tree: classification and regression tree classifier. We use the Gini coefficient as the measure of node impurity and arbitrarily set a minimum number of 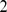 instances per leaf.
- NB: naive Bayes classifier. We employ a Gaussian function to estimate the likelihood of each onset deviation.
- LR: logistic regression classifier. We use L2-regularized logistic regression with automatically-scaled intercept fit.
- SVM: support vector machine. We consider a linear kernel (SVM-L) and a radial basis function kernel (SVM-R).
- Random: random classifier. We additionally consider a random classifier as the baseline. It outputs a randomly selected class from the pool of all available training labels.

### Evaluation Strategy

For each data set 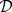 we perform standard 20-times, 10-fold, out-of-sample cross-validation [30]–[32]. Even if our music collection is already balanced (10 performances per piece), we force internal training and testing data sets to be balanced as well. Hence, we train with 9 performances per piece and test with 1. We additionally ensure that all classifiers observe the same training/testing sets. As different selections of 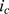 could affect the results, we repeat the whole process 100 times, in order to obtain a reliable estimation of all possible accuracies (not only for average accuracies and their standard deviations, but also to have a proper idea of maximum/minimum values and reliably assessing statistical significance). In summary, we generate 100 data sets 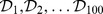 and test each classifier with them. This yields a total of 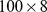 accuracy values (100 for each classifier, including the random baseline) computed from 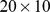 folds.

As we use matched samples 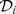, we assess statistical significance with the well-known Wilcoxon signed-rank test [33]. The Wilcoxon signed-rank test is a non-parametric statistical hypothesis test used when comparing two matched samples (or related samples, or repeated measurements) in order to assess whether their population mean ranks differ. It is the natural alternative to the Student's 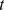-test for dependent samples when the population distribution cannot be assumed to be normal [33]. We use as input the 200 accuracy values obtained for one classifier and the random baseline. To compensate for multiple pairwise comparisons, we apply the Holm-Bonferroni method [34], a post-hoc statistical analysis method controlling the so-called family-wise error rate that is more powerful than the usual Bonferroni correction [49].

## Supporting Information

Figure S1
**Scatter plot of relative note durations from the score versus onset deviations.** This plot corresponds to a random sample of 50 values per performance. Different colors correspond to different compositions. Kendall 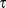 rank correlation coefficients between relative note durations 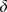 and onset deviations 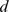 were low across all possible 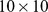 comparisons between score and performance: 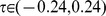, 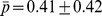.(TIF)

Figure S2
**Scatter plot of note intervals from the score Δ versus onset deviations 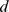.** This plot corresponds to a random sample of 50 values per performance. Different colors correspond to different compositions. Kendall 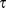 rank correlation coefficients between note intervals **Δ** and onset deviations 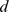 were low across all possible 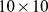 comparisons between score and performance: 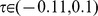, 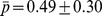.(TIF)

Figure S3
**Onset deviation distributions and long-range correlations.** (A) Examples of onset deviation distributions 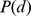. For comparison we also depict a standard Gaussian distribution (see MM) with mean and standard deviation directly derived from 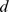 (Table 3). (B) Examples of power spectral densities 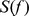 from the full onset deviation sequences. The visual aids correspond to a power law as formulated in MM. From left to right, the power law exponents obtained are 1, 0.8, 0.7, and 0.4. Frequencies 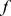 are linearly scaled for ease of visualization. For both plots, the color-coded legends correspond to recording identifiers, CXXPYY, where XX corresponds to composition number, XX 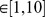, and YY corresponds to performance number, YY 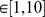.(TIF)

Figure S4
**Classification accuracy as a function of the length of the onset deviation sequence when shuffling.** The error bars correspond to the standard deviation and the shaded area denotes the range of all possible values (including minimum and maximum). The visual aid corresponds to a constant straight line of the form 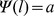. In the plot 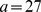.(TIF)

Figure S5
**Average classification accuracy as a function of the number of compositions.** Results obtained using a sequence length 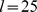. The error bars correspond to the standard deviation and the shaded area corresponds to the range of all possible values (including minimum and maximum). The visual aids correspond to a power law of the form 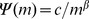, where 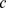 is a constant, 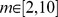 is the number of compositions, and 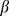 is the power law exponent. The upper one is plotted with 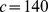 and 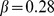, and is associated with classification accuracies. The lower one is plotted with 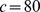 and 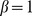, and corresponds to the random baseline. The exponent associated with classification accuracies is much smaller than the one for the random baseline, what suggests that the absolute difference between the two increases with the number of considered compositions and, therefore, with the size of the data set.(TIF)

Figure S6
**Classification accuracy as a function of the length of the onset deviation sequences: comparison between onset deviations and relative note durations.** These are KNN-E (A), KNN-D (B), Tree (C), NB (D), LR (E), SVM-L (F), and SVM-R (G). Dark blue squares correspond to onset deviation sequences 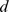, light orange diamonds correspond to relative note durations 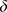, and black dashed lines correspond to the random baseline.(TIF)

Figure S7 **Semi-automatic onset detection accuracy.** (A) Histogram of analyzed onset temporal differences. (B) Onset deviation error rate as a function of a threshold (see text).
(TIF)

Figure S8
**Onset placement and imputation example.** (A) After synchronizing the audio with the score, we have matches for all score onsets except 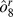. (B) For this, we look at possible onset candidates inside the green window, inside which the highest amplitude peak is highlighted (see text).(TIF)

Table S1 **Information about compositions.** The last two columns correspond to note durations relative to the beat (see text).
(PDF)

Table S2 **Information about compositions, musicians, and recordings.** Table relating composers, compositions, and recordings. Columns correspond to compositions except for the last one, which corresponds to performer birth and death dates. Rows correspond to performers. In each cell, recording year and recording label are shown.
(PDF)

Table S3 **Summary statistics for onset deviations for all performances of a given composition. All values are given in seconds.**
(PDF)
