# Supplementary figures and images for: Note Onset Deviations as Musical Piece Signatures
Source: PLoS One. 2013 Jul 31;8(7):e69268. doi: 10.1371/journal.pone.0069268 (PMC3729570; doi:10.1371/journal.pone.0069268)

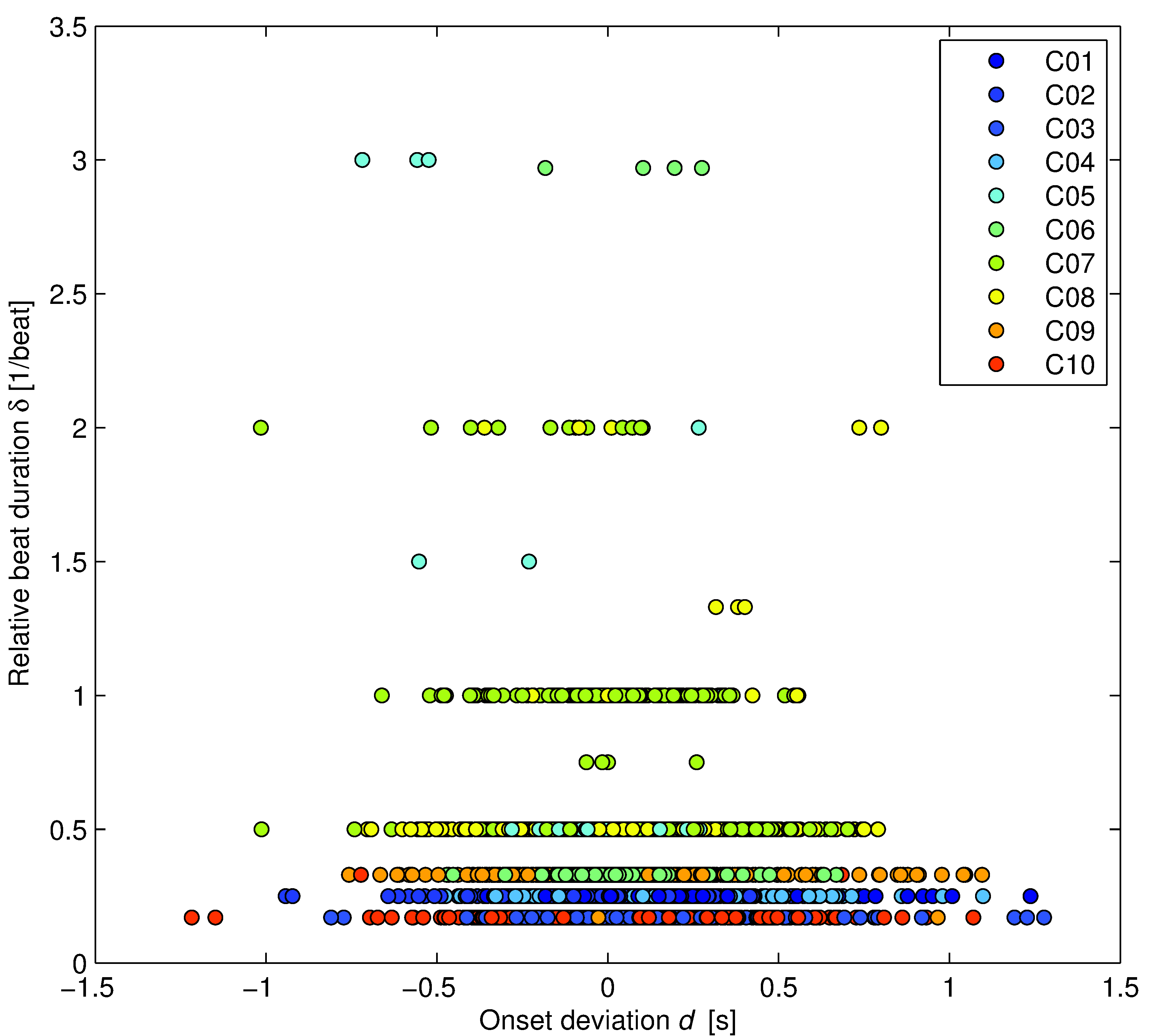

Supplement: Figure S1 — Scatter plot of relative note durations from the score versus onset deviations. This plot corresponds to a random sample of 50 values per performance. Different colors correspond to different compositions. Kendall rank correlation coefficients between relative note durations and onset deviations were low across all possible comparisons between score and performance: , . (TIF) [file pone.0069268.s001.tif]

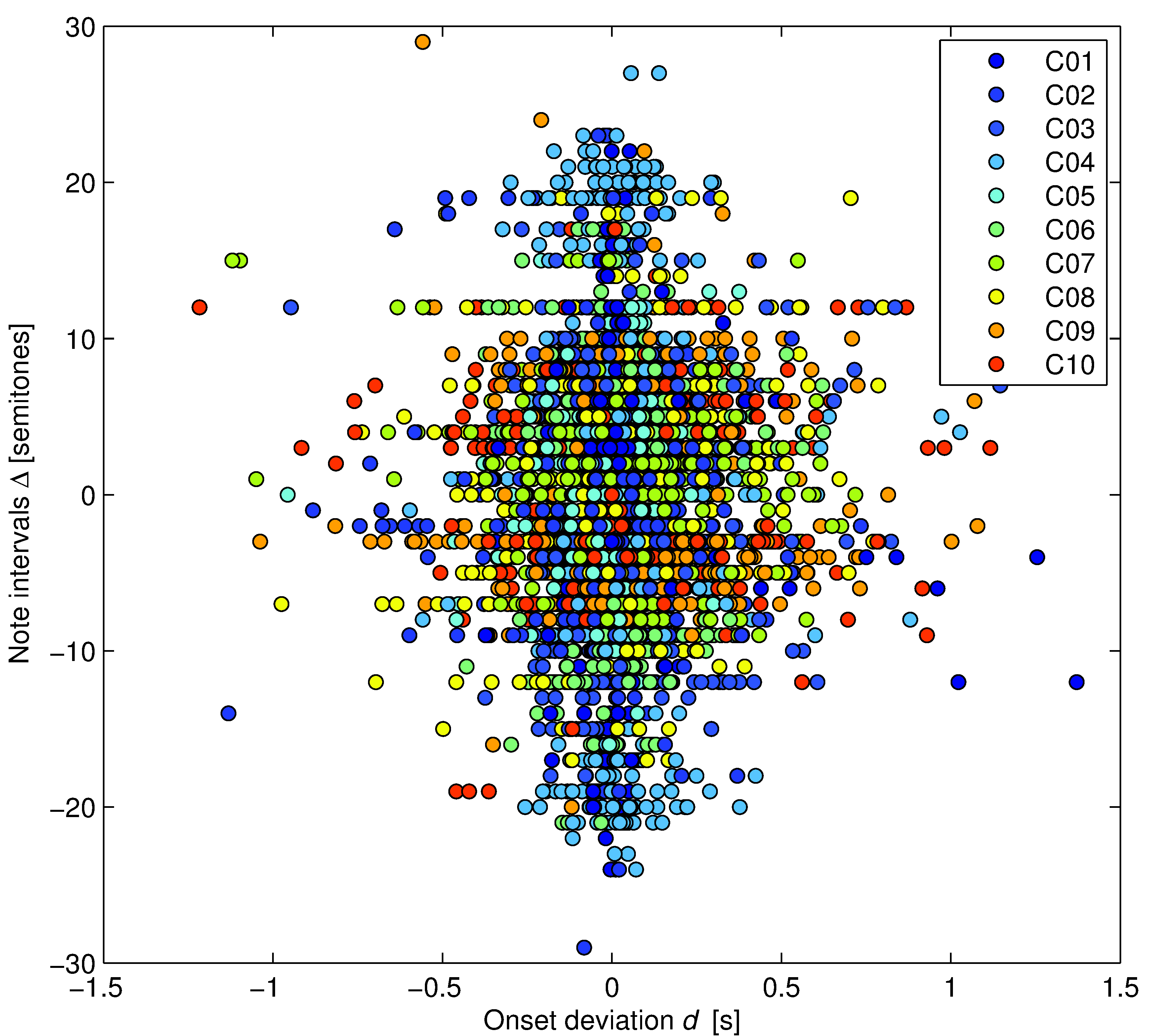

Supplement: Figure S2 — Scatter plot of note intervals from the score Δ versus onset deviations . This plot corresponds to a random sample of 50 values per performance. Different colors correspond to different compositions. Kendall rank correlation coefficients between note intervals Δ and onset deviations were low across all possible comparisons between score and performance: , . (TIF) [file pone.0069268.s002.tif]

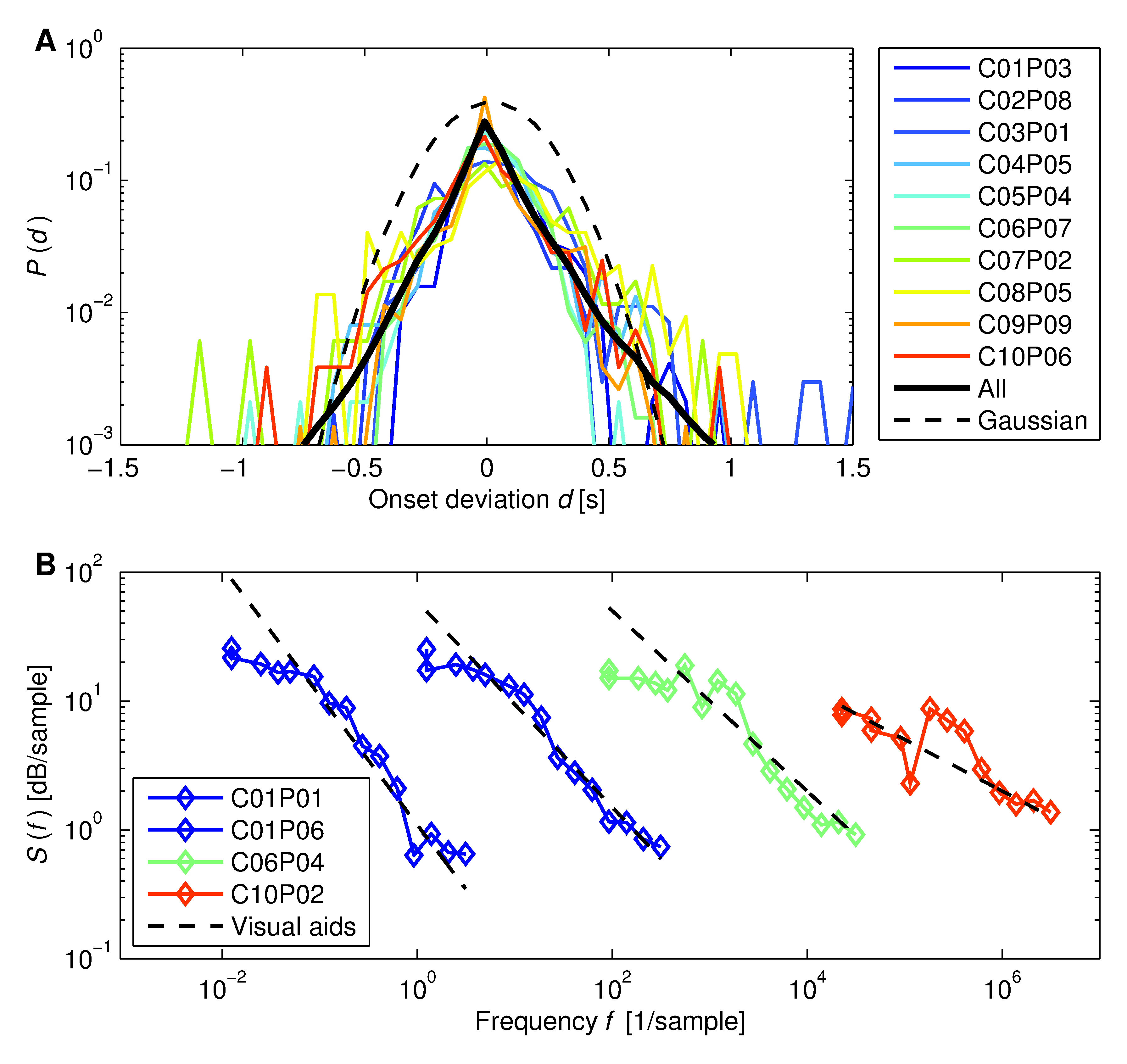

Supplement: Figure S3 — Onset deviation distributions and long-range correlations. (A) Examples of onset deviation distributions . For comparison we also depict a standard Gaussian distribution (see MM) with mean and standard deviation directly derived from (Table 3). (B) Examples of power spectral densities from the full onset deviation sequences. The visual aids correspond to a power law as formulated in MM. From left to right, the power law exponents obtained are 1, 0.8, 0.7, and 0.4. Frequencies are linearly scaled for ease of visualization. For both plots, the color-coded legends correspond to recording identifiers, CXXPYY, where XX corresponds to composition number, XX , and YY corresponds to performance number, YY . (TIF) [file pone.0069268.s003.tif]

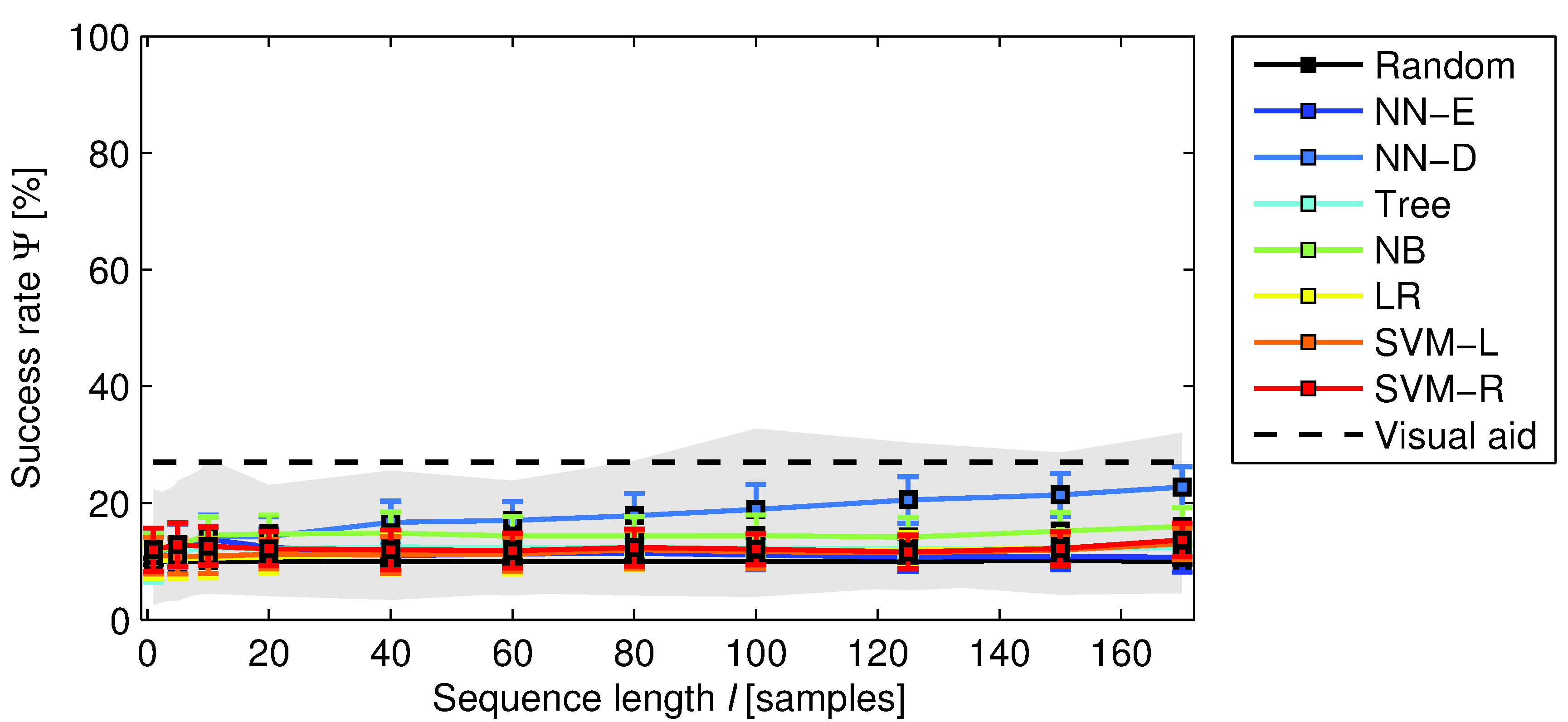

Supplement: Figure S4 — Classification accuracy as a function of the length of the onset deviation sequence when shuffling. The error bars correspond to the standard deviation and the shaded area denotes the range of all possible values (including minimum and maximum). The visual aid corresponds to a constant straight line of the form . In the plot . (TIF) [file pone.0069268.s004.tif]

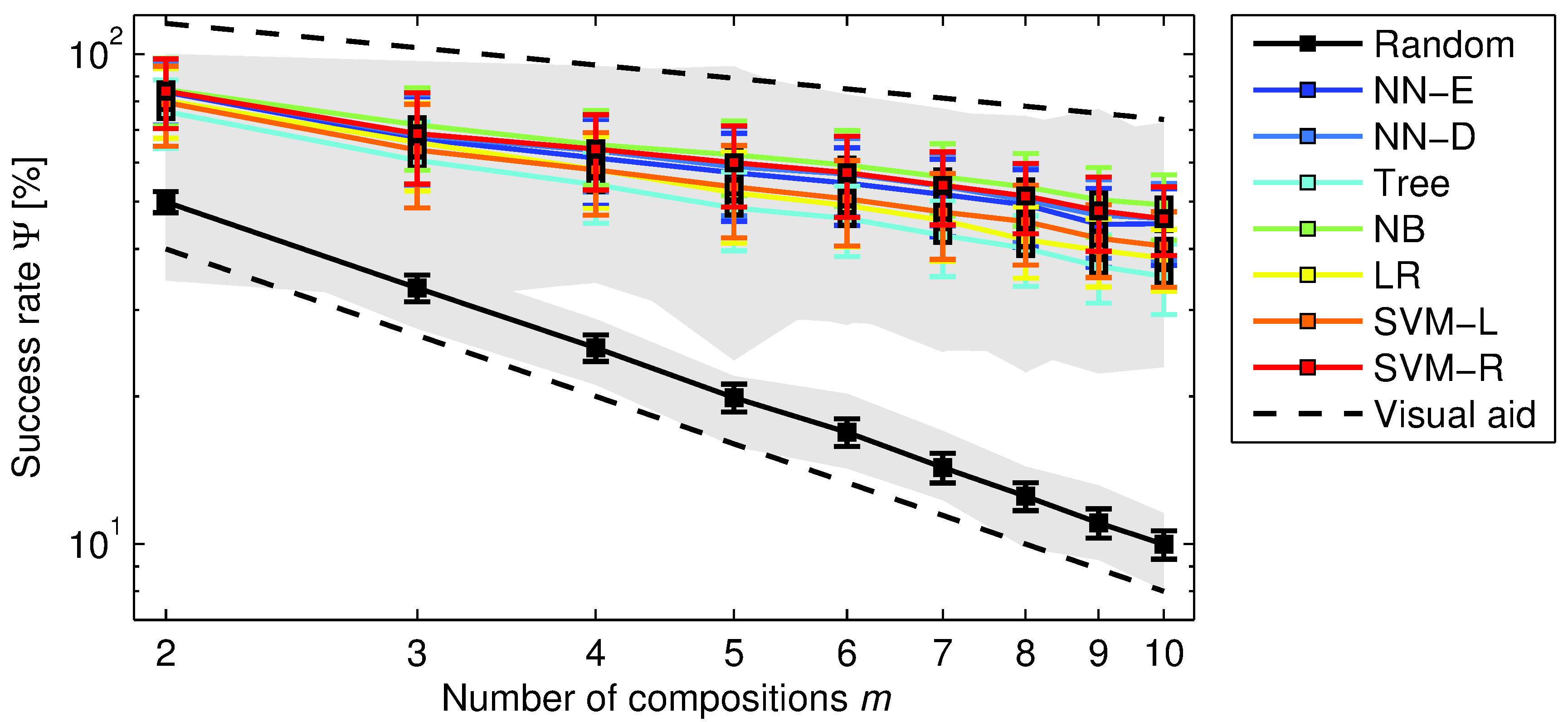

Supplement: Figure S5 — Average classification accuracy as a function of the number of compositions. Results obtained using a sequence length . The error bars correspond to the standard deviation and the shaded area corresponds to the range of all possible values (including minimum and maximum). The visual aids correspond to a power law of the form , where is a constant, is the number of compositions, and is the power law exponent. The upper one is plotted with and , and is associated with classification accuracies. The lower one is plotted with and , and corresponds to the random baseline. The exponent associated with classification accuracies is much smaller than the one for the random baseline, what suggests that the absolute difference between the two increases with the number of considered compositions and, therefore, with the size of the data set. (TIF) [file pone.0069268.s005.tif]

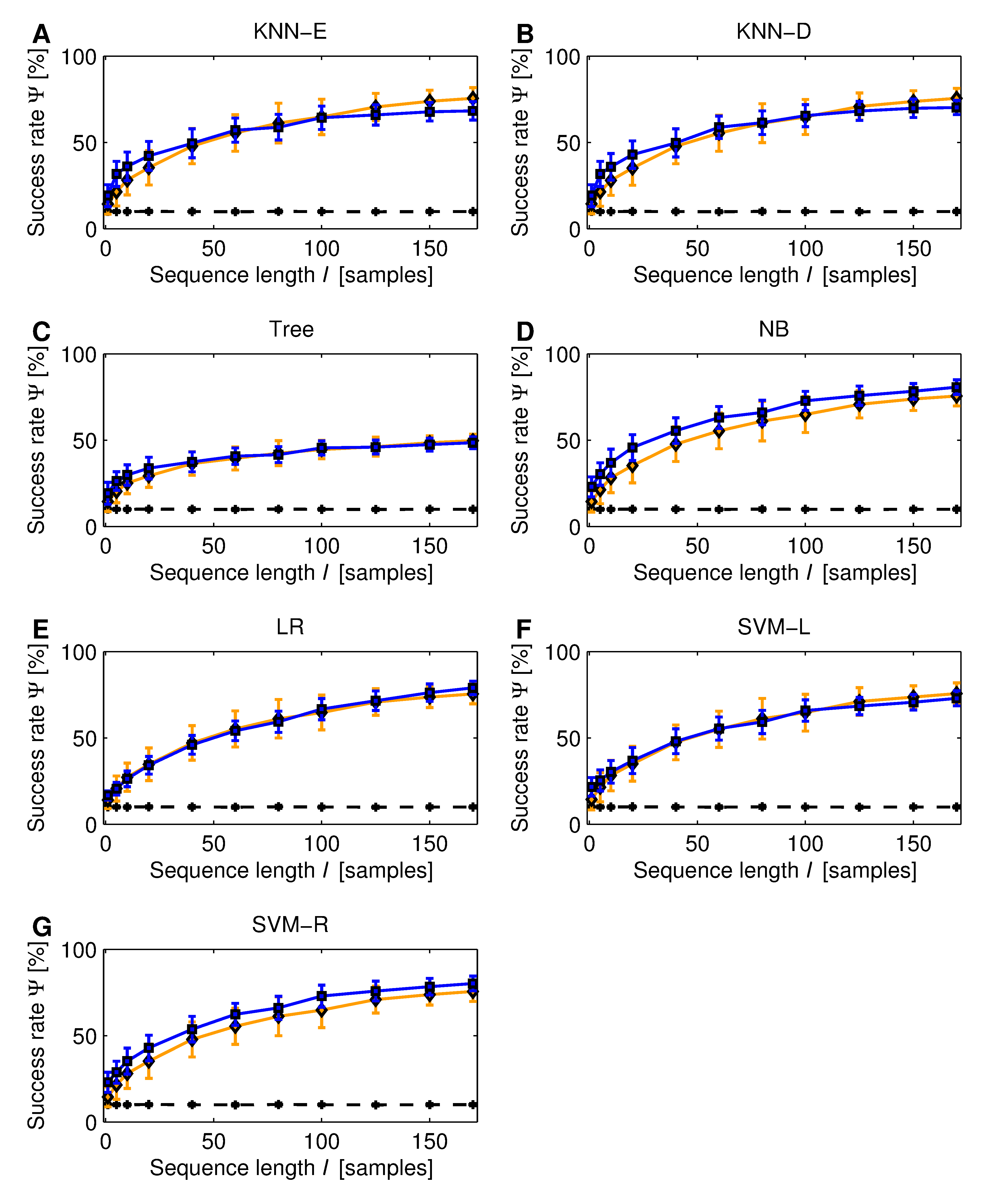

Supplement: Figure S6 — Classification accuracy as a function of the length of the onset deviation sequences: comparison between onset deviations and relative note durations. These are KNN-E (A), KNN-D (B), Tree (C), NB (D), LR (E), SVM-L (F), and SVM-R (G). Dark blue squares correspond to onset deviation sequences , light orange diamonds correspond to relative note durations , and black dashed lines correspond to the random baseline. (TIF) [file pone.0069268.s006.tif]

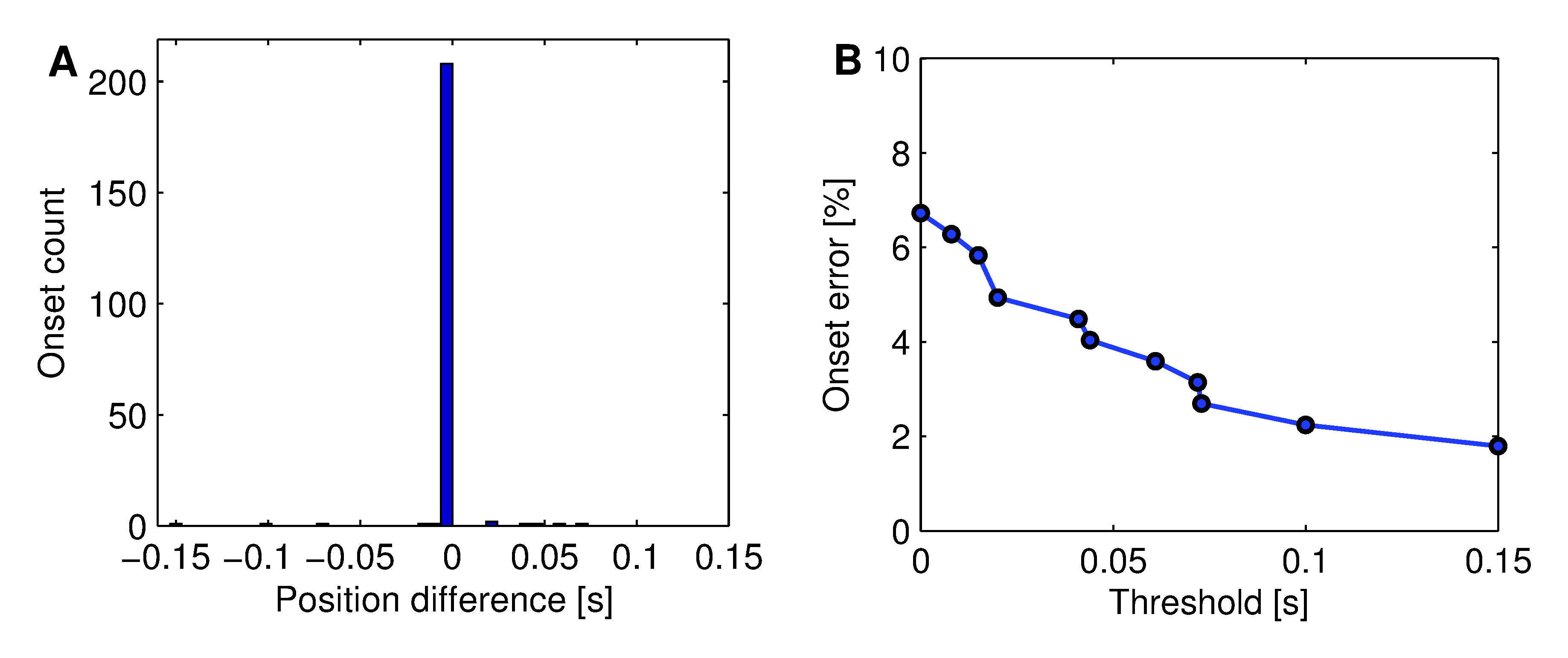

Supplement: Figure S7 — Semi-automatic onset detection accuracy. (A) Histogram of analyzed onset temporal differences. (B) Onset deviation error rate as a function of a threshold (see text). (TIF) [file pone.0069268.s007.tif]

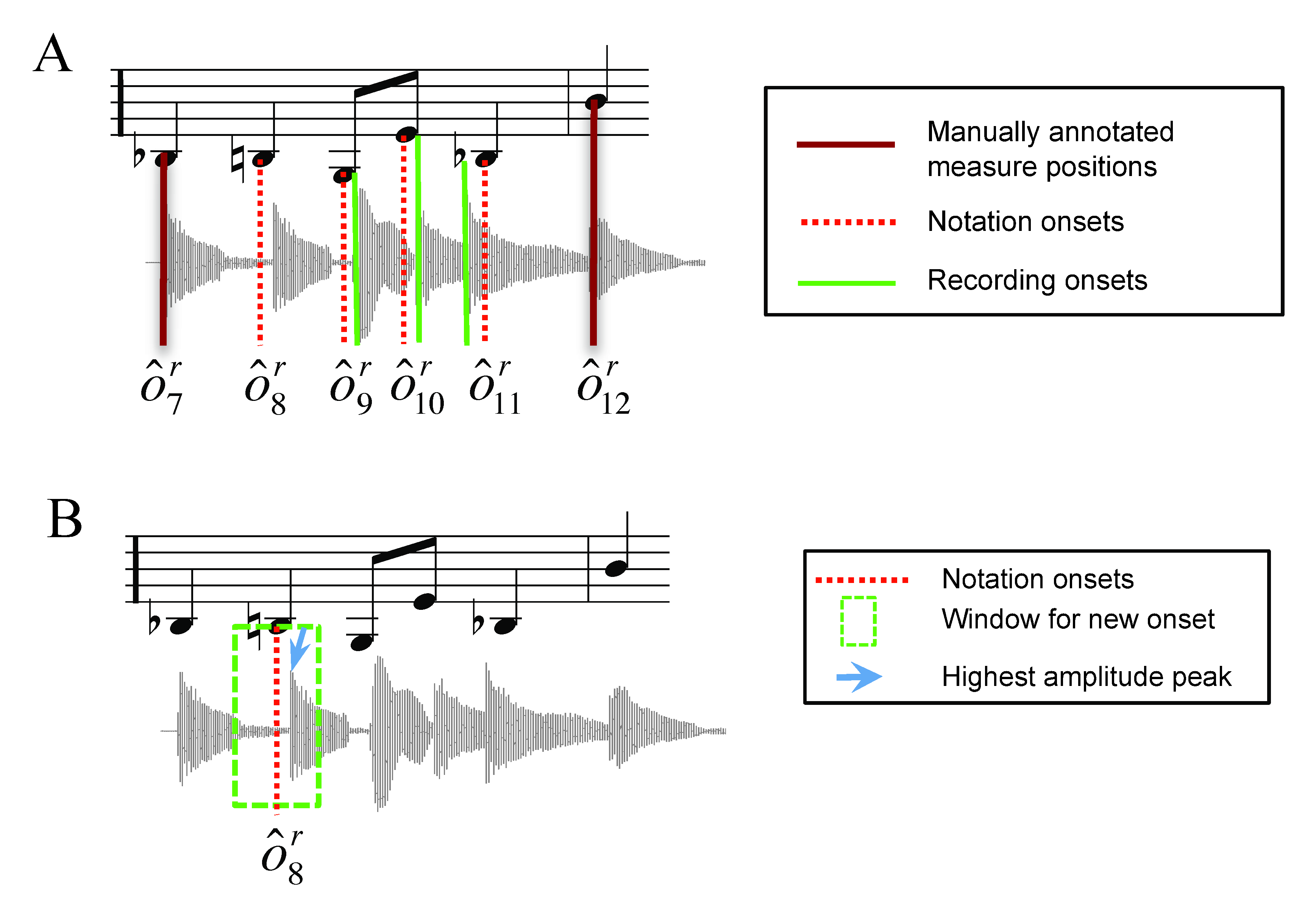

Supplement: Figure S8 — Onset placement and imputation example. (A) After synchronizing the audio with the score, we have matches for all score onsets except . (B) For this, we look at possible onset candidates inside the green window, inside which the highest amplitude peak is highlighted (see text). (TIF) [file pone.0069268.s008.tif]
