# Supplementary material for: Note Onset Deviations as Musical Piece Signatures
Source: PLoS One. 2013 Jul 31;8(7):e69268. doi: 10.1371/journal.pone.0069268 (PMC3729570; doi:10.1371/journal.pone.0069268)
Supplement: Table S1 — Information about compositions. The last two columns correspond to note durations relative to the beat (see text). (PDF) [file pone.0069268.s009.pdf]

ID	Composition	Composer	Composition year	Historical period	Tempo	Number of notes	Shortest note	Longest note
**C01**	BWV 999	J.S. Bach	1720	Baroque	Allegretto	505	1/4	3
**C02**	BWV 1007	J.S. Bach	1720	Baroque	Andante	641	1/4	2
**C03**	La Catedral, Prelude	A. Barrios	1921	Modern	Lento	379	1/4	2
**C04**	C minor Prelude	A. Barrios	1920	Modern	Moderato	361	1/6	1
**C05**	Cavatina	S. Myers	1970	Modern	Andante	515	1/8	3
**C06**	Romance	Anonymous	ca. 1800	Classical	Andante	680	1/3	2
**C07**	Adelita	F. Tarrega	ca. 1880	Romantic	Moderato	173	1/4	2
**C08**	Lagrima	F. Tarrega	ca. 1880	Romantic	Andante	219	1/8	2
**C09**	Moonlight Sonata	L.V. Beethoven	1801	Classical	Adagio	794	1/3	4
**C10**	Etude B minor	F. Sor	1828	Romantic	Allegretto	27 9	1/2	2
