# Supplementary material for: Note Onset Deviations as Musical Piece Signatures
Source: PLoS One. 2013 Jul 31;8(7):e69268. doi: 10.1371/journal.pone.0069268 (PMC3729570; doi:10.1371/journal.pone.0069268)
Supplement: Table S2 — Information about compositions, musicians, and recordings. Table relating composers, compositions, and recordings. Columns correspond to compositions except for the last one, which corresponds to performer birth and death dates. Rows correspond to performers. In each cell, recording year and recording label are shown. (PDF) [file pone.0069268.s010.pdf]

Note Onset Deviations as Musical Piece Signatures

Joan Serra, Tan Hakan Özaslan, and Josep Lluis Arcos

	J.S. Bach	J.S. Bach	A.Barrios	Anonymous	F. Tarrega	S. Myers	F. Tarrega	A. Barrios	L.V. Beethoven	F. Sor		
	BWV 1007	BWV 999	Catedral, Prelude	Romance	Lagrima	Cavatina	Adelita	C Min. Prelude	Moonlight Sonat	Etude BMin.	Born	Death
Alexandre Pier Federici			2011-AndanteMusic								-	-
Ana Vidovic						2007-YouTube					1988	Alive
Andrea Gasperi			1996-ScreenStuio								1964	Alive
Andrei Krylov	2009 - AndreiKrylov							2007-AndreiKrylovMusic			1959	Alive
Andres Segovia		1965 - Bravo! Records								1962-MCAClassics	1893	1987
Andrew Schulman		1989-CentaurRecords									1960	Alive
Angel Romero						1990-Telarc		2001-Delos	1976-AngelRecords		1946	Alive
Bob Fetherolf		2008-FullSailMusic									-	-
Cary Greisch							2001-BellaMusic				1958	Alive
Cesar Amaro					2009-YouTube						1948	2012
Chandra Rajagopal							2009-Youtube				1987	Alive
Christoper Parkening						2007-AngelRecords					1947	Alive
Craig Ogden						2010-X5MusicGroup					1988	Alive
Cristiano Porqueddu			2009-BrilliantClassics								1975	Alive
Dan Hopson				2011-DanHopson							1950	Alive

Note Onset Deviations as Musical Piece Signatures

Joan Serra, Tan Hakan Özaslan, and Josep Lluis Arcos

	J.S. Bach	J.S. Bach	A.Barrios	Anonymous	F. Tarrega	S. Myers	F. Tarrega	A. Barrios	L.V. Beethoven	F. Sor		
	**BWV 1007**	**BWV 999**	**Catedral, Prelude**	**Romance**	**Lagrima**	**Cavatina**	**Adelita**	**C Min. Prelude**	**Moonlight Sonat**	**Etude BMin.**	**Born**	**Death**
**Daniele Magli**										2010-YouTube	1960	Alive
**Danny Masters**			2010-DannyMasters								1976	Alive
**Eduardo Fernandez**		1989-DeccaMusicGrou pLimited									1952	Alive
**Edward Trybek**			2007-EdwardTrybek								1982	Alive
**Eric Henderson**								2009-YouTube			1958	Alive
**Erling Moldrup**				2011-Muzart							1943	Alive
**Eros Roselli**										2008-YouTube	1966	Alive
**Filomena Moretti**			2010-Transart								1973	Alive
**Franciscus Terpstra**								2001-Franciscus Terpstra			1949	Alive
**Gareth Koch**		1997-AustralianBroadc astingCorporatio n									1962	Alive
**Gerry Johnston**				2011-GerryJohnston							1950	Alive
**Goran Sollicher**	1992-DeutscheGramm ophonGmbH									1998-DeutscheGramm ophonGmbH	1955	Alive
**Irina Kulikova**	2011-Naxos										1979	Alive
**Jean Jacques Fimbel**									2010-JeanJacque Fimbel		1955	Alive
**Jen Chi Encin**							x				1966	Alive
**Jerome Ducharme**							2005-YouTube				1978	Alive

Note Onset Deviations as Musical Piece Signatures

Joan Serra, Tan Hakan Özaslan, and Josep Lluis Arcos

	J.S. Bach	J.S. Bach	A.Barrios	Anonymous	F. Tarrega	S. Myers	F. Tarrega	A. Barrios	L.V. Beethoven	F. Sor		
	BWV 1007	BWV 999	Catedral, Prelude	Romance	Lagrima	Cavatina	Adelita	C Min. Prelude	Moonlight Sonat	Etude BMin.	Born	Death
Joe						2010-YouTube					1980	Alive
John Demans					x						-	-
John H. Clarke								2007-UrbanTribe Production			1982	Alive
John Q										2009-YouTube	-	-
John Williams	1958-IDIS						1995-Sony				1941	Alive
Jonas Lefvert						2011-YouTube					1981	Alive
Jonathan Adams		1998-SonicGrapefruit			1998-SonicGrapefruit				1998-SonicGrapefruit		1961	Alive
Joseph Sullinger									2008-EroicaClassical Recordings		1969	Alive
Juanillo De Alba				2011-CountdownMedia GmbH							1952	Alive
Julian Bream										2006-YouTube	1933	Alive
Kevin McCormick			2007-MiralisRecords	2004-MirablisRecords	2004-MirablisRecords				2004-MirablisRecords		1968	Alive
Lianto Tjahjoputro							2009-YouTube				1963	Alive
Liona Boyd					2004-MostonRecords				2004-MostonRecords		1962	Alive
Luigi Attademo		2009-BrilliantClassics									1972	Alive
Manuel Barrueco		2004-BigJoKeMusic	2005-EMI								1952	Alive
Marcelo Kayath									2009-MusicalConcepts		1964	Alive

Note Onset Deviations as Musical Piece Signatures

Joan Serra, Tan Hakan Özaslan, and Josep Lluis Arcos

	J.S. Bach	J.S. Bach	A.Barrios	Anonymous	F. Tarrega	S. Myers	F. Tarrega	A. Barrios	L.V. Beethoven	F. Sor		
	BWV 1007	BWV 999	Catedral, Prelude	Romance	Lagrima	Cavatina	Adelita	C Min. Prelude	Moonlight Sonat	Etude BMin.	Born	Death
Michael Lucarelli								2006-YouTube			1959	Alive
Michel Fiorelli							2010-Youtube				1961	Alive
Milos Karadaglic					2011-DeutscheGrammophonGmbH				2011-DeutscheGrammophonGmbH		1982	Alive
Narciso Yepes										2003-DeutscheGrammophonGmbH	1960	Alive
Nelson Amos				2008-NelsonAmas							1952	Alive
Pascal Beausseron							2012-YouTube				1970	Alive
Per Olov Kindgren										2007-YouTube	1956	Alive
Pere Salicru				2010-Edivox							1964	Alive
Pete Downes	2007-WiserProduction										1952	Alive
Raoulle Sansfaon							2010-YouTube				-	-
Rey De La Torre					1947-Classical Monuments						1917	1994
Ricardo Prieto	2009-RicardoPrieto										1973	Alive
Richard Ames	2009-YouTube										1965	Alive
Robert Westaway	2005-BluePebbleMusic	2005-BluePebbleMusic		2005-BluePebbleMusic	2005-BluePebbleMusic				2005-BluePebbleMusic		1985	Alive
Rodrigo Escoba								2011-TrackMusic			-	-
Rodrigo Lorente				2012-NatTeamMedia							-	-

Note Onset Deviations as Musical Piece Signatures

Joan Serra, Tan Hakan Özaslan, and Josep Lluis Arcos

	J.S. Bach	J.S. Bach	A.Barrios	Anonymous	F. Tarrega	S. Myers	F. Tarrega	A. Barrios	L.V. Beethoven	F. Sor		
	BWV 1007	BWV 999	Catedral, Prelude	Romance	Lagrima	Cavatina	Adelita	C Min. Prelude	Moonlight Sonat	Etude BMin.	Born	Death
Roger Lurel							2011-YouTube				1969	Alive
Sean Kelly	2007-OpeningDay Entertainment	2007-OpeningDay Entertainment			2007-OpeningDay Entertainment	2007-OpeningDay Entertainment					1958	Alive
Sharon Isbin		2002-WarnerClassics									1956	Alive
Simon Dinnigan						2009-Sony					1968	Alive
Soren Bodker Madsen										1998-Barbarossa	1956	Alive
Stanley Myers						2001-Milan Entertainment					1930	1993
Susan McDonald			1995-MayflyRecords								1965	Alive
Taylor Jones	2011-LionGroup Records										-	-
Terry Muska									2011-TalkingTacoMusic		1946	Alive
Tim Hall								2008-YouTube			1965	Alive
Tom Tilley				2012-CustodianRecord s	2012-CustodianRecord s						1965	Alive
Tom Ward						2011-YouTube					1983	Alive
Vicente Covers			2010-Naxos								1982	Alive
YouTube Unknown Performer 1										2010-YouTube	-	-
YouTube Unknown Performer 2								x			-	-
YouTube Unknown Performer 3								x			-	-
