# Supplementary material for: Note Onset Deviations as Musical Piece Signatures
Source: PLoS One. 2013 Jul 31;8(7):e69268. doi: 10.1371/journal.pone.0069268 (PMC3729570; doi:10.1371/journal.pone.0069268)
Supplement: Table S3 — Summary statistics for onset deviations for all performances of a given composition. All values are given in seconds. (PDF) [file pone.0069268.s011.pdf]

	C01	C02	C03	C04	C05	C06	C07	C08	C09	C10
**Mean**	0.043	-0.004	0.031	0.031	-0.004	0.014	0.038	0.018	0.024	0.024
**Standard deviation**	0.164	0.220	0.192	0.182	0.124	0.148	0.259	0.232	0.260	0.259
**Maximum anticipation**	-0.420	-2.584	-0.947	-0.597	-1.288	-0.621	-1.401	-0.975	-1.220	-1.217
**Maximum delay**	2.225	0.864	1.616	1.360	0.573	1.026	0.950	1.262	1.636	1.363
